# Coronary artery bypass grafting vs. percutaneous coronary intervention in coronary artery disease patients with advanced chronic kidney disease: A Chinese single-center study

**DOI:** 10.3389/fsurg.2022.1042186

**Published:** 2023-01-20

**Authors:** Yang Li, Xuejian Hou, Xiaoyu Xu, Zhuhui Huang, Taoshuai Liu, Shijun Xu, Hongliang Rui, Jubing Zheng, Ran Dong

**Affiliations:** ^1^ Department of Cardiac Surgery, Beijing Anzhen Hospital, Capital Medical University, Beijing, China; ^2^ Department of Pharmacy, Beijing Anzhen Hospital, Capital Medical University, Beijing, China

**Keywords:** coronary artery bypass grafting, percutaneous coronary intervention, chronic kidney disease, coronary heart disease, renal insufficiency

## Abstract

**Objectives:**

Aims to compare the contemporary and long-term outcomes of coronary artery bypass grafting (CABG) and percutaneous coronary intervention (PCI) in coronary artery disease (CAD) patients with advanced chronic kidney disease (CKD).

**Methods:**

823 CAD patients with advanced CKD (eGFR < 30 ml/min/1.73 m^2^) were collected, including 247 patients who underwent CABG and 576 patients received PCI from January 2014 to February 2021. The primary endpoint was all-cause death. The secondary endpoints included major adverse cardiac and cerebrovascular events (MACCEs), myocardial infarction (MI), stroke and revascularization.

**Results:**

Multivariable Cox regression models were used and propensity score matching (PSM) was also performed. After PSM, the 30-day mortality rate in the CABG group was higher than that in the PCI group but without statistically significant (6.6% vs. 2.4%, *p *= 0.24). During the first year, patients referred for CABG had a hazard ratio (HR) of 1.42 [95% confidence interval (CI), 0.41–3.01] for mortality compared with PCI. At the end of the 5-year follow-up, CABG group had a HR of 0.58 (95%CI, 0.38–0.86) for repeat revascularization, a HR of 0.77 (95%CI, 0.52–1.14) for survival rate and a HR of 0.88(95%CI, 0.56–1.18) for MACCEs as compared to PCI.

**Conclusions:**

Among patients with CAD and advanced CKD who underwent CABG or PCI, the all-cause mortality and MACCEs were comparable between the two groups in 30 days, 1-year and 5 years. However, CABG was only associated with a significantly lower risk for repeat revascularization compared with PCI at 5 years follow-up.

## Introduction

The incidence of coronary artery disease (CAD) is increasing by years, the probability of CAD patients combined with advanced chronic kidney disease (CKD) (eGFR < 30 ml/min/1.73 m^2^) is higher than 50% (1). Cardiovascular disease is one of the leading causes of death in patients with advanced CKD (2). Renal insufficiency increases the risk of death after revascularization (3). At present, coronary artery bypass grafting (CABG) and percutaneous coronary intervention (PCI) are the main options for treating patients with coronary artery disease involving multiple vessels or left main stenosis (4).

CKD patients have complicated etiologies (such as hypertension and diabetes) and severe coronary lesions. Although many comparative studies have proven that CABG has a better long-term prognosis in CKD patients than PCI, prospective random controlled trials are still lacking (5, 6). Most of the studies were conducted in Western or some other Asian countries like Japan and Korea, it was scarce in China because of the late performance for the cardiac surgery since the 1990s. As one of the largest hospitals for the treatment of cardiovascular diseases in China, our center has the capacity to accomplish nearly 20,000 revascularization operations each year and can provide abundant clinical resources for the coronary revascularization.

The present study retrospectively analyzed the clinical data of advanced CKD patients who underwent CABG or PCI in our hospital and completed short and long-term follow-up, aiming to compare the effect of the two revascularization methods and improve the prognosis.

## Methods

### Study participants

From January 2014 to February 2021, a total of 823 CAD patients with advanced CKD (eGFR < 30 ml/min/1.73 m^2^) were treated in our hospital, including 247 patients who underwent CABG (CABG group) and 576 patients who received PCI (PCI group). Patients who had nephrectomy, kidney transplantation, and combined with valve or other cardiac surgical procedure were excluded. The diagnoses of all patients were confirmed by coronary angiography, including angina, non-ST-segment elevation myocardial infarction(MI) and ST-segment elevation MI. Clinical data were collected, including preoperative baseline data and intraoperative and postoperative data.

### Data collection

Baseline data included age, body mass index (BMI), sex, eGFR, dialysis, blood urea nitrogen (BUN), hemoglobin (Hb), family history of coronary heart disease, comorbidities such as hypertension or diabetes mellitus, smoking history, prior PCI, New York Heart Association (NYHA) classification, left ventricular ejection fraction (LVEF), the number of narrowed coronary arteries and left main coronary artery disease. The surgical data included surgical approach, surgery time, number of coronary artery anastomoses, number of stents, and intra-aortic balloon pump (IABP) usage. Postoperative data included in-hospital mortality, perioperative MI, cerebral infarction, new-onset atrial fibrillation (AF), discharge medications, hospitalization time and cost. The mechanical ventilation time, ICU care time and the rate of undergoing thoracotomy for hemostasis were collected in the CABG group. However, the rate of coronary perforation, pericardial tamponade or conversion to emergency CABG were collected in the PCI group only. The conventional surgical method was applied for patients in the PCI group. The stents used included drug-eluting and bare-metal stents. The surgical procedures for CABG included off-pump coronary artery bypass grafting (OPCABG) and on-pump coronary artery bypass grafting (On-pump CABG).

### Study end points

The primary endpoint was all-cause deaths. Secondary endpoints included major adverse cardiac and cerebrovascular events (MACCEs), stroke, recurrent MI and repeat revascularization. All follow-up results were obtained by phone or mail from patients themselves or their relatives.

### Statistical analysis

For propensity score matching (PSM), the matching conditions included demographic data included sex, age, BMI and surgical-related factors such as eGFR, BUN, Hb, LVEF, hypertension and diabetes. Age, BMI, LVEF, eGFR and Hb were continuous variables; sex, hypertension, diabetes, stroke, prior AF, and left main coronary artery (LMCA) disease were categorical variables. Logistic regression analysis was used to establish the CABG propensity score, which was then used for 1:1 matching with the PCI group. When the count data of the two groups followed a normal distribution, the T-test of paired samples was used for analysis; the values were expressed as the mean ± standard deviation. When the data of the two groups did not conform to a normal distribution, the rank sum test of paired samples was applied; the values were expressed as the median. The measurement data were analyzed with the McNemar's test and are expressed as frequencies and percentages. Effects of PCI compared to CABG for individual end points were expressed as HRs with 95% confidence intervals (CIs). All analyses were conducted by a statistician with the use of SAS software version 9.4 (SAS Institute Inc). All reported *p* values were 2-sided, and *p* values < 0.05 were regarded as statistically significant.

## Results

### Baseline clinical and procedural characteristics before PSM

The CABG group had a higher percentage of males, family history of CAD, Hb level, prior MI or carotid artery stenosis than the PCI group (*p *< 0.05). However, the CABG group had lower eGFR or serum cholesterol level than the PCI group (*p *< 0.05). The number of narrowed coronary arteries in CABG group was higher than that in the PCI group (*p *< 0.01). The preoperative left ventricular end-diastolic diameter in the CABG group was higher than that in the PCI group (*p *< 0.05), while there was no significant difference in LVEF between the two groups (*p *= 0.41) (Table 1). In the CABG group, the rate of dialysis was 27.9%, the rate of left internal mammary artery usage (LIMA) and OPCABG ratio were 91.1% and 89.9%, respectively. The average coronary anastomosis was 3.0 ± 0.8. In the PCI group, the rate of dialysis was 24.5%, the rate of drug-eluting stent (DES) usage was approximately 98.1%, and the average number of implanted stents was 2.6 ± 1.1 (Table 2). Compared with the PCI group, the CABG group had higher in-hospital mortality rate (9.3% vs. 1.7%, *p *< 0.001) and higher incidences of perioperative MI and new-onset AF (18.6% vs. 11.8%, *p *= 0.009; 20.6% vs. 2.8%, *p *< 0.001). In the CABG group, the ICU care time was 60.8 ± 58.8 h, the ventilator support time was 45.6 ± 42.9 h, and the rate of IABP usage was 12.6%. In the PCI group, the proportion of intraoperative complications including coronary artery dissection, perforation or pericardium tamponade were 2.3% (Tables 2, 3).

**Table 1:** Comparison of preoperative baseline characteristics between CABG and PCI groups.

Clinical variables	Before PS matched		Standard difference	*p*-Value	After PS matched		Standard difference	*p*-Value
	CABG (*n* = 247)	PCI (*n* = 576)			CABG (*n* = 166)	PCI (*n* = 166)		
Age (years)	64.1 ± 9.3	63.5 ± 12.0	0.06	0.44	63.9 ± 9.6	64.4 ± 11.6	−0.04	0.70
Male sex (%)	199 (80.6)	381 (66.1)	0.33	<.001	128 (77.1)	123 (74.1)	0.07	0.50
BMI (kg/m^2^)	25.5 ± 3.2	26.2 ± 11.6	0.09	0.15	25.5 ± 3.3	25.2 ± 3.2	0.09	0.40
Family history (%)	14 (5.7)	7 (1.2)	0.25	0.002	6 (3.6)	4 (2.4)	0.07	0.53
Hypertension (%)	196 (79.4)	492 (85.4)	−0.16	0.03	130 (78.3)	137 (82.5)	−0.11	0.36
Diabetes mellitus (%)	100 (40.5)	250 (43.4)	−0.06	0.44	65 (39.2)	70 (42.2)	−0.06	0.58
Smoking (%)	99 (40.1)	191 (33.2)	0.14	0.06	60 (36.1)	58 (34.9)	0.03	0.83
Carotid artery stenosis (%)	43 (17.5)	20 (3.5)	0.57	<.001	12 (7.2)	11 (6.6)	0.02	0.74
NSTEMT (%)	42 (17.0)	117 (20.3)	−0.09	0.27	29 (17.5)	34 (20.5)	−0.08	0.48
STEMI (%)	23 (9.3)	77 (13.4)	−0.13	0.10	16 (9.6)	20 (12.0)	−0.08	0.48
Unstable angina pectoris (%)	164 (66.4)	346 (60.1)	0.13	0.09	108 (65.1)	102 (61.4)	0.08	0.49
Emergency procedure (%)	2 (0.8)	13 (2.3)	−0.12	0.15	1 (0.6)	3 (1.8)	−0.11	0.32
Prior MI (%)	104 (42.1)	188 (32.6)	0.20	0.01	61 (36.7)	59 (35.5)	0.03	0.81
Prior PCI (%)	38 (15.4)	99 (17.2)	0.08	0.68	26 (15.7)	23 (13.9)	0.05	0.65
Prior atrial fibrillation (%)	13 (5.3)	41 (7.1)	−0.08	0.32	8 (4.8)	14 (8.4)	−0.15	0.20
Prior TIA or stroke (%)	31 (12.6)	76 (13.2)	−0.02	0.80	18 (10.8)	21 (12.7)	−0.06	0.61
Hemoglobin level (g/L)	111.3 ± 20.3	111.7 ± 23.1	−0.02	0.81	112.4 ± 21.0	110.5 ± 23.8	0.08	0.45
Albumin (mmol/L)	38.8 ± 6.0	41.8 ± 9.1	0.39	<.001	39.1 ± 5.7	40.9 ± 9.2	0.49	0.08
Triglyceride (mmol/L)	2.3 ± 1.5	2.3 ± 2.0	−0.01	0.93	2.2 ± 1.3	2.1 ± 1.9	0.03	0.74
Cholesterol (mmol/L)	4.4 ± 1.2	4.7 ± 1.3	−0.20	0.01	4.5 ± 1.2	4.7 ± 1.4	−0.18	0.09
eGFR (ml/min/1.73 m^2^)	8.3 ± 3.3	10.2 ± 4.5	−0.47	<.001	8.7 ± 3.2	9.0 ± 4.4	−0.07	0.51
Dialysis (%)	69 (27.9)	141 (24.5)	0.12	0.30	44 (26.5)	40 (24.1)	0.07	0.57
Calcium (mmol/L)	2.1 ± 0.4	2.1 ± 0.2	−0.03	0.75	2.2 ± 0.4	2.1 ± 0.2	0.16	0.15
Left main stenosis (%)	32 (13.0)	51 (8.9)	0.14	0.07	24 (14.5)	23 (13.9)	0.02	0.87
**No. of narrowed**								
Coronary arteries	3.1 ± 0.79	2.5 ± 0.98	0.81	<.001	2.9 ± 0.78	3.0 ± 0.99	−0.11	0.22
1 (%)	11 (4.4)	94 (16.3)			9 (5.4)	10 (6)		
2 (%)	31 (12.6)	201 (34.9)			38 (22.9)	25 (15.1)		
3 (%)	141 (57.1)	206 (35.8)			75 (45.2)	102 (61.4)		
≥4 (%)	64 (25.9)	75 (13.0)			44 (26.5)	29 (17.5)		
LVEDD (mm)	52.9 ± 6.7	51.3 ± 7.2	0.23	<.05	52.1 ± 6.8	52.2 ± 7.1	−0.02	0.84
Ventricular aneurysm (%)	17 (6.9)	53 (9.2)	−0.09	0.27	11 (6.6)	18 (10.8)	−0.15	0.16
LVEF (%)	55.4 ± 10.4	54.7 ± 11.9	0.06	0.41	54.8 ± 0.8	56.2 ± 12.0	−0.12	0.28
NYHA classification (%)			0.29	0.02			0.23	0.16
1	12 (4.9)	41 (7.1)			7 (4.2)	15 (9.0)		
2	136 (55.1)	363 (63.0)			98 (59.0)	95 (57.2)		
3	81 (32.8)	118 (20.5)			50 (30.1)	41 (24.7)		
4	18 (7.3)	54 (9.4)			11 (6.6)	15 (9.0)		

**Table 2:** Comparison of perioperative data between CABG and PCI groups.

Procedural variables	Before PS matched		After PS matched	
	CABG (*n* = 247)	PCI (*n* = 576)	CABG (*n* = 166)	PCI (*n* = 166)
Duration of operation (h)	4.3 ± 1.0	–	4.2 ± 1.0	–
Number of grafts	3.0 ± 0.8	–	2.9 ± 0.7	–
Number of stents	–	2.6 ± 1.1		2.9 ± 1.2
LIMA usage (%)	225 (91.1)	–	152 (91.6)	–
OPCABG (%)	222 (89.9)	–	150 (90.4)	–
Drug-eluting stent usage (%)	–	565 (98.1)	–	163 (98.2)
Coronary artery dissection (%)	–	4 (0.7)	–	2 (1.2)
Coronary perforation (%)	–	5 (0.9)	–	3 (1.8)
Pericardium tamponade (%)	–	4 (0.7)	–	3 (1.8)

**Table 3:** Comparison of postoperative data between CABG and PCI groups.

Postoperative variables	Before PS Matched		Standard difference	*p*-Value	After PS Matched		Standard difference	*p*-Value
	CABG (*n* = 247)	PCI (*n* = 576)			CABG (*n* = 166)	PCI (*n* = 166)		
ICU time (h)	60.8 ± 58.8	–			61.4 ± 58.7	–		
Mechanic ventilation time (h)	45.6 ± 42.9	–			45.4 ± 41.3	–		
IABP (%)	31 (12.6)	18 (3.1)	0.56	<.001	23 (13.8)	6 (3.6)	0.61	<0.001
Red Blood cell transfusion (U)	3.4 ± 2.8	–			3.5 ± 2.9	–		
Reoperation for bleeding (%)	21 (8.5)	–			13 (7.8)	–		
Re-intubation (%)	12 (4.9)	–			9 (5.4)	–		
Wound complications (%)	12 (4.9)	–			8 (4.8)	–		
Tracheotomy (%)	10 (4.0)	–			6 (3.6)	–		
Myocardial infarction (%)	46 (18.6)	68 (11.8)	0.19	0.009	29 (17.5)	20 (12.0)	0.15	0.16
Cerebral infarction (%)	2 (0.8)	11 (1.9)	−0.10	0.25	1 (0.6)	2 (1.2)	−0.06	0.56
Infection (%)	16 (6.5)	10 (1.7)	0.24	<.001	9 (5.4)	4 (2.4)	0.16	0.17
New-onset AF (%)	51 (20.6)	16 (2.8)	0.58	<.001	42 (25.3)	7 (4.2)	0.62	<.0001
Cost (USD)	18909 ± 12599	8876 ± 4521	1.07	<.001	18230 ± 10421	10035 ± 5244	0.99	<.0001
In-hospital mortality (%)	23 (9.3)	10 (1.7)	0.26	<.001	11 (6.6)	4 (2.4)	0.13	0.24
**Medication at discharge (%)**								
Aspirin	220 (96.5)	544 (96.3)	0.01	0.89	153 (96.8)	159 (98.1)	−0.08	0.65
Clopidogrel	181 (79.3)	510 (90.3)	−0.61	0.002	127 (76.5)	143 (88.3)	−0.51	<.0001
Beta-blockers	199 (87.3)	514 (91.0)	−0.12	0.12	136 (86.1)	144 (88.9)	−0.09	0.32
Statin	171 (75.0)	528 (93.5)	−0.52	<.001	122 (77.2)	153 (94.4)	−0.51	<.0001
Nitrates	196 (86.0)	423 (74.9)	0.28	<.001	137 (86.7)	128 (79.0)	0.21	0.14

### Procedural characteristics and early outcomes after PSM

166 patients were-well matched in each group (Table 1). In the CABG group, the mean operative time was 4.3 ± 1.0 h, the average number of graft was 2.9 ± 0.7, and the OPCABG ratio was 90.4%. In the PCI group, the average number of stents was 2.9 ± 1.2. In the CABG group, the percentage of patients who underwent postoperative thoracotomy for hemostasis was 7.8%, the wound infection rate was 4.8%, and the proportion of patients who underwent tracheotomy was 3.6%. Compared with the PCI group, the CABG group had a higher incidence of new-onset AF (25.3% vs. 4.2%, *p *< 0.001), more support of IABP (13.8% vs. 3.6%, *p *< 0.001) and higher treatment costs (18230 ± 10421 vs. 10035 ± 5244$, *p *< 0.001). The two groups exhibited no significant differences in in-hospital mortality and the incidence of perioperative complications such as MI (6.6% vs. 2.4%, *p *= 0.24; 17.5% vs. 12.0%, *p *= 0.16) (Tables 2, 3).

### Long-term outcomes before PSM

In the CABG group, the 1-year and 5-year survival rates were 92.7% and 72.9%, respectively; the freedom from revascularization were 95.1% and 82.6%, respectively; and the freedom from MACCEs were 85.8% and 62.7%, respectively. In the PCI group, the 1-year and 5-year survival rates were 93.4% and 66.9%, respectively; the freedom from revascularization were 92.8% and 75.9%, respectively; and the freedom from MACCEs were 88.0% and 60.8%, respectively.

### Long-term outcomes after PSM

**Figure 1:**
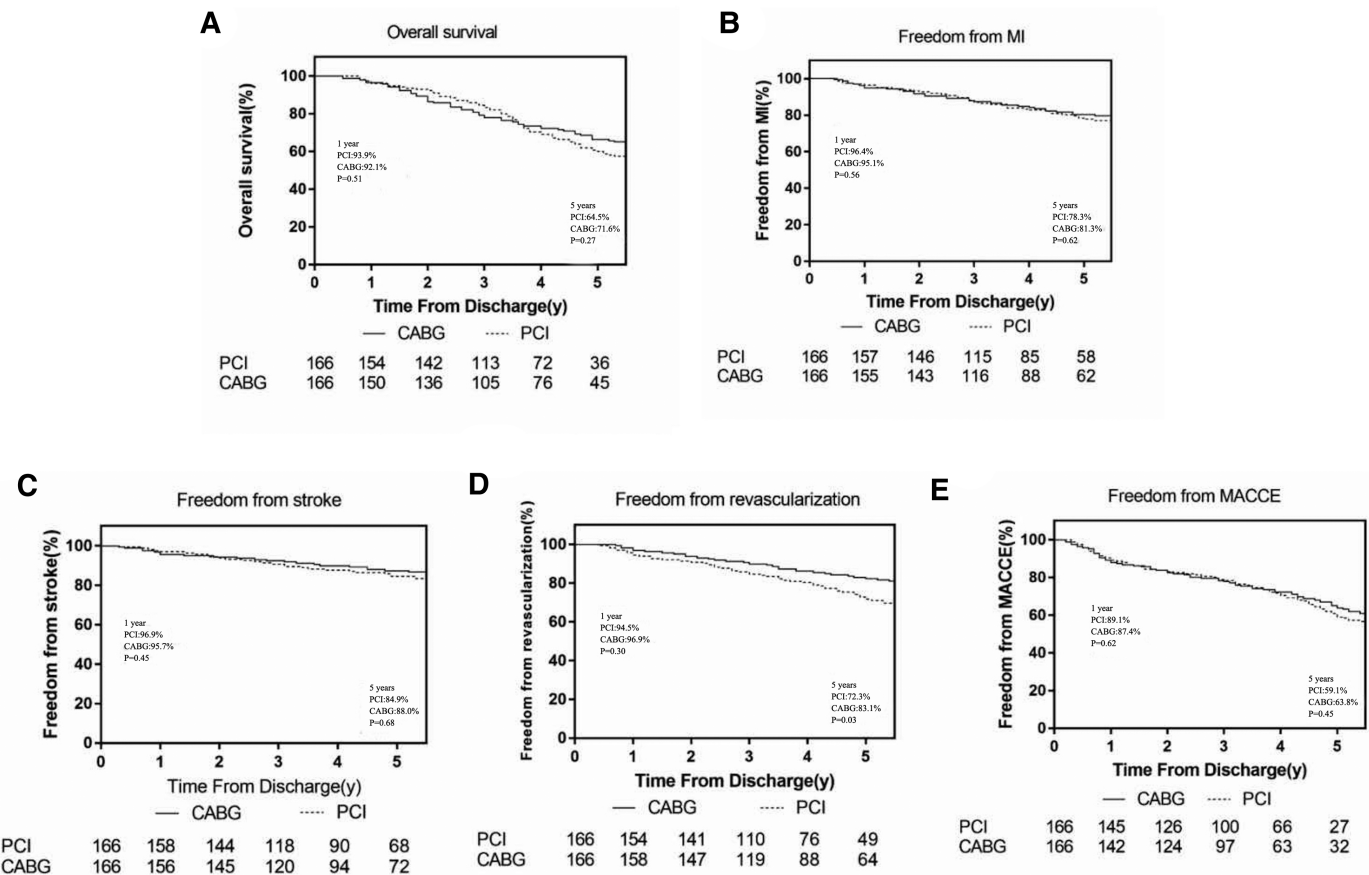
The comparison of survival and MACCEs at 1-year and 5-years between the two groups.

In the CABG group, the 1-year and 5-year survival rates were 92.1% and 71.6%, respectively; the freedom from revascularization were 96.9% and 83.1%, respectively; the freedom from MACCEs were 87.4% and 63.8%, respectively; the freedom from MI were 95.1% and 81.3%, respectively and the freedom from stroke were 95.7% and 88%, respectively. In the PCI group, the 1-year and 5-year survival rates were 93.9% and 64.5%, respectively; the freedom from revascularization were 94.5% and 72.3%, respectively; the freedom from MACCEs were 89.1% and 59.1%, respectively; the freedom from MI were 96.4% and 78.3%, respectively and the freedom from stroke were 96.9% and 84.9%, respectively (Figure 1).

**Figure 2:**
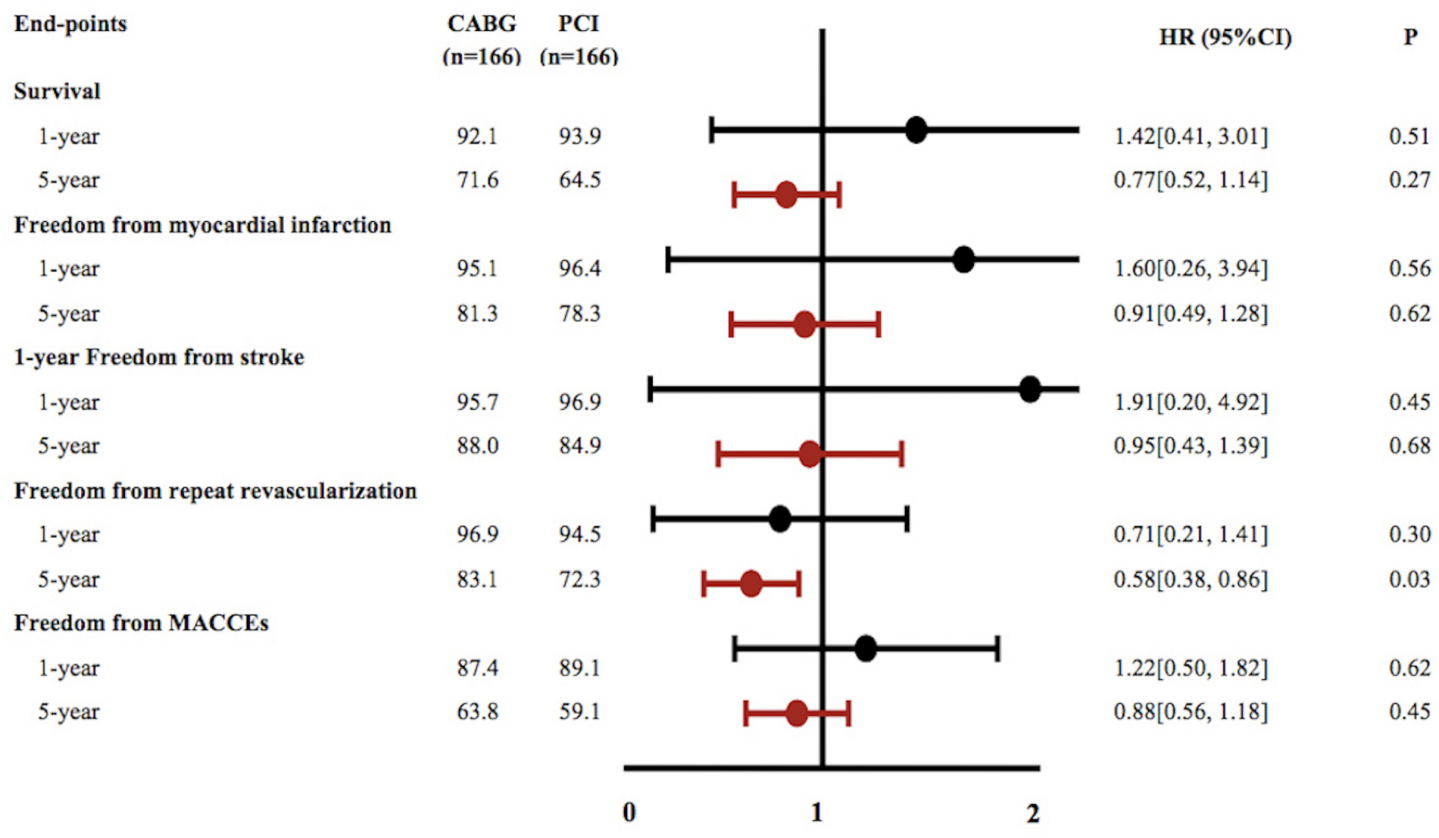
Cumulative event curves for outcomes of survival, freedom from myocardial infarction, freedom from stroke, freedom from repeat revascularization and freedom from major adverse cardiovascular and cerebrovascular events (**A–E**). Curves generated using the Kaplan–Meier approach Forest plot showing associations for different endpoints. Hazard ratios and 95% confidence intervals generated from Cox proportional hazards regression without covariate adjustment. Black squares and lines show point estimates and 95% CI for patients with PCI while Red squares and lines reflect associations for patients with CABG.

During the 1-year follow-up, patients referred for CABG had a slight non-significant increase in the hazard of mortality (HR 1.42; 95%CI, 0.41–3.01, *p *= 0.51), MACCEs (HR 1.22; 95%CI, 0.50–1.82, *p *= 0.62), recurrent MI (HR 1.60; 95%CI, 0.26–3.94, *p *= 0.56) and stroke(HR 1.91; 95%CI, 0.20–4.92, *p *= 0.45) compared with PCI, while the PCI group had higher incidence of repeat revascularization (HR 0.71; 95%CI, 0.21–1.41, *p *= 0.30). At the end of the 5-year follow-up, CABG was associated with significantly lower risks for repeat revascularization compared with PCI, (HR 0.58; 95%CI, 0.38–0.86; *p *= 0.03). Furthermore, the CABG group had a higher survival rate (HR 0.77; 95%CI, 0.52–1.14, *p *= 0.27), higher freedom from MACCEs (HR 0.88; 95%CI 0.56–1.18, *p* = 0.45), higher freedom from recurrent MI (HR 0.91; 95%CI, 0.49–1.28, *p *= 0.62) and freedom from stroke (HR 0.95; 95%CI, 0.43–1.39, *p* = 0.68) than those in the PCI group but without statistically significant (Figure 2).

## Discussion

Cardiovascular disease is one of the most common complications in CKD patients and is also the major cause of death (1). According to the United States Renal Data System (USRDS), the cardiac mortality accounts for approximately 45% (7). It is increasingly recognized that patients with advanced CKD who underwent revascularization had worse outcomes than that with normal renal function. However, there are lack of international guidelines for revascularization therapy for CKD patients in the worldwide.

In our research, patients in the CABG group were relatively young and the male patients accounted for the majority; the proportion of emergency operations was relatively low, the preoperative cardiac function and size were nearly normal and OPCABG accounted for nearly 90%. The low risk factors and the good preoperative baseline status could have reduced the mortality rates and incidences of complications. There are some studies on the analysis of risk factors for CABG mortality in advanced CKD patients, Li et al. (8) reported 134 cases of dialysis patients who underwent CABG and found that age, history of cerebral stenosis and emergency surgery were risk factors for death. Gautam R. Shroff (9) reported that age over 65 years, white race, peritoneal dialysis and heart failure were independent risk factors for death. ASCERT (10) indicated that the risk factors for death include age, chronic obstructive pulmonary disease, cerebrovascular diseases, low EF, female, preoperative IABP, CPB, combined surgery, CCS/NYHA grade IV and incomplete vascularization.

The risk of surgery for advanced CKD patients is higher than that for general patients. Valentino Bianco et al. (11) reported that dialysis patients had a higher operative (30-day) mortality (8.6%), higher blood transfusion rate, higher rate of ventilator use for more than 24 h, higher incidence of sternal wound infection, second thoracotomy and new-onset AF. Rahmanian et al. (12) found that the mortality rate for dialysis patients undergoing cardiac surgeries was 3.9 times (12.7%) that for general patients and that dialysis was a risk factor for in-hospital death, which may be due to preoperative dysregulation of blood calcium and phosphorus, abnormal blood lipids and platelet metabolism, and severe coronary artery calcification in ESRD patients. In this study, the in-hospital mortality rate (9.3%) for the CABG group and the incidence of perioperative complications were roughly similar to that previously reported in western countries.

For CAD patients with advanced CKD, it is still controversial whether CABG or PCI should be chosen for revascularization. A meta-analysis (13) included twelve studies demonstrate that in dialysis patients, CABG was associated with long-term survival but a higher risk for early mortality, and the risk for repeat revascularization was higher with PCI. Another study (14) reporting higher risk of mortality, MI, and repeat revascularization in the PCI arm compared with CABG. Chung Hee Baek (15) reported that the CABG group had fewer MACCEs than the DES group but that the overall survival rate did not differ. Manabe S (16) reported that the MACCE-free survival rate and angina-free survival rate were significantly higher in patients receiving CABG surgery than in those receiving PCI. Terazawa et al. (17) analyzed 125 dialysis patients who underwent CABG and PCI found that revascularization with CABG was superior to DES. Akira Marui (3) reported that PCI and CABG exhibited no significant difference in all-cause death; however, the PCI group had a higher risk of revascularization than the CABG group. In 2014, ESC/EACTS reported that CABG was superior to PCI for treating CAD patients with moderate to severe renal diseases (18). In the present study, we found that the two groups exhibited no significant differences at the end of the 1-year follow-up. But during the end of the 5-year follow-up, the CABG group had a lower risk of revascularization, a better trend of prognosis than the PCI group, indicating that the treatment efficacy of CABG was better than that of PCI.

The long-term survival rate of the CABG group in our study was slightly higher than that reported abroad. Gaudam R. Shroff et al. (9) reported that the 1- and 5-year survival rates in CABG were 70% and 28%, while the DES group had 1- and 5-year survival rates of 71% and 24%, respectively. Leontyev et al. (19) analyzed 483 dialysis patients who underwent CABG and found that the 2, 4, and 6-year survival rates were 64.1%, 42.2%, and 30.6%, respectively. Our finding might be related to the following reasons. First, in our study, the patients had better preoperative biochemical indicators, such as hemoglobin level, albumin, and had nearly normal blood lipid levels. Anemia can lead to a series of pathophysiological changes, resulting in reduced quality of life and decreased patient survival. High albumin is a nutritional status and inflammation marker for dialysis patients. The CABG group in this study had hemoglobin levels of 111.29 ± 20.27 g/dl and albumin levels of 38.83 ± 6.02 g/dl, were better that those of patients in the US (20). Second, the mainly procedure we used was OPCABG. The avoidance of extracorporeal circulation reduced the need for blood transfusions, the release of inflammatory mediators and shortened ventilator assistance and ICU care time. For patients with reduced cardiac function, the timely use of IABP and extracorporeal circulation can ensure the safety of complete revascularization and surgery. Third, the increased survival rate may be related to the race of the patients. Rangrass et al. (21) found that the quality of life of different ethnic groups after CABG treatment varies greatly. For the Asian population, Marui et al. (3) reported that patients with three-vessel disease or left main artery disease have a better 5-year mortality rate, MI rate and revascularization rate after CABG than after PCI. Previous studies also reported that white race is a risk factor for death (10).

The reported mortality rates for dialysis (6) patients in different countries and different regions vary. The Chinese National Renal Data System (CNRDS) reported dialysis patient mortality rates in China in 2007, 2008, 2009 and 2010 of 7.4%, 7.6%, 9.0% and 8.6%, respectively (22), lower than those reported in the United States and Japan. The possible reason might be that the dialysis patients in China are generally in good condition, and the average age is relatively low. The most common disease is primary glomerular diseases, while in American patients, renal failure is mainly related to diabetes and hypertension (7). Furthermore, Chinese dialysis patients mostly receive treatment in hospitals, and the contact between doctors and patients is conducive to improving patient compliance and improving long-term prognosis.

The incidence of recent and long-term revascularization in CABG group was significantly better than PCI patients, this might be attributable to several factors. First, CABG treatment can provide complete revascularization, however in PCI procedure culprit vessels were always firstly handled. Second, transit time flow measurement was universally used during bypass surgery to ensure long-term patency. Third, preoperative blood glucose and blood lipid levels in patients with CABG were lower than PCI. The increased blood glucose or lipid levels may reduce the patency rate of the graft or the stent.

Recently, ISCHEMIA-CKD study (23) reported that PCI did not reduce the risk of death or nonfatal myocardial infarction in patients with moderate to severe myocardial ischemia compared with drug therapy. However, this study focused on patients with moderate myocardial ischemia (61.4%), with an average follow-up time of only 2.2 years. According to our study, long-term follow-up shows that CABG could improve the prognosis of advanced CKD patients with severe myocardial ischemia, which needs more RCT studies to confirm.

This study has some limitations. First, the data are from a single center and cannot represent the overall situation in China. Second, this is a retrospective study, and there may be selection bias; even PSM cannot completely eliminate bias. Third, there is still a lack of clear revascularization guidelines for CKD or dialysis patient. Finally, the sample size was relatively small, which may affect the applicability of the conclusions and the accuracy of the efficacy analysis. In addition, long-term follow-up results require further analysis. Finally, the timing for CABG and PCI can be different, making it a longitudinal nature of the intervention. Some patients may also have several PCI during the follow up periods, this can be technically difficult to perform. Future studies addressing these limitations will be necessary in the future.

## Conclusion

In this cohort of 823 CAD patients with advanced CKD undergoing coronary revascularization, treatment with CABG or PCI exhibited similar all-cause mortality and MACCE in 30 days, 1 year and the whole follow-up period. However, CABG was associated with an extremely decrease in the risk of repeat revascularization compared to PCI at 5 years. Although the outcomes were equivalent to PCI, CABG might provide a possible trend towards benefit for the long-term prognosis. In future research, we will expand the sample size, prolong the follow-up duration to obtain more reliable results to guide the clinical.

## Data Availability

The raw data supporting the conclusions of this article will be made available by the authors, without undue reservation.
